# Ebstein Anomaly and Right Aortic Arch in Patient with Charge Syndrome

**DOI:** 10.3390/medicina57111239

**Published:** 2021-11-13

**Authors:** Inguna Lubaua, Madara Teraudkalna

**Affiliations:** 1 Department of Pediatrics, Riga Stradins University, LV-1007 Riga, Latvia; Madara.Teraudkalna@gmail.com; 2 Clinic for Pediatric Cardiology and Cardiac Surgery, Children’s Clinical University Hospital, LV-1004 Riga, Latvia

**Keywords:** Ebstein anomaly, right aortic arch, congenital heart disease, charge syndrome

## Abstract

Ebstein anomaly is a rare congenital heart disease characterized by a varying degree of anatomical and functional abnormalities of tricuspid valve and right ventricle. It often coexists with other congenital cardiac malformations. Up to 79–89% of patients with Ebstein anomaly have interatrial communication in the form of patent oval foramen or atrial septal defect and more than one-third has other types of cardiac malformations. Association between Ebstein anomaly and right aortic arch is extremely rare and only few cases have been described in the literature so far. Much rarer than with other cardiac malformations, Ebstein anomaly is associated with non-cardiac malformations or genetic syndromes. Several cases of association between Ebstein anomaly and Charge syndrome have been reported, nevertheless, Ebstein anomaly accounts for less than 1% of cardiac defects seen in patients with Charge syndrome. In this case report, we present a unique case of a patient with Charge syndrome where both Ebstein anomaly and right aortic arch are present. The diagnosis of Ebstein anomaly and right aortic arch was established prenatally. In the first years of life, the patient did not exhibit any remarkable symptoms. However, over time, deterioration of right ventricle function and increased tricuspid regurgitation were observed, requiring consideration of surgical treatment at the age of five. In addition, delay in physical, motor, and mental development was observed and thus, at the age of five, the patient was consulted by a medical geneticist and a gene panel to test for structural heart defects was ordered. The test showed a mutation in chromodomain helicase DNA binding protein 7 (CHD7) gene, which, along with clinical features, allowed to establish a diagnosis of Charge syndrome. To the best of the authors’ knowledge, this is the first case report of a patient with Charge syndrome, Ebstein anomaly, and right aortic arch that has been described in the literature.

## 1. Introduction

Ebstein anomaly is a rare congenital heart disease, comprising only around 1% of all congenital heart diseases and has an estimated incidence of around 0.5–0.7 in 10,000 live births [1,2,3]. It is primarily characterized by a displacement of abnormal tricuspid valve towards the apex of the right ventricle. The posterior and septal leaflets of the tricuspid valve are attached to the wall of the right ventricle, while the anterior leaflet of the valve retains some attachment to the valve ring. The anterior leaflet usually is redundant, sail-like, may contain fenestrations, and, in some cases, may cause outflow tract obstruction. The abnormal displaced tricuspid valve causes downward placement of the functional annulus and splits the right ventricle into two parts. The upper part known as the atrialized part due to a continuous communication with the right atrium and the lower part consisting of a normal ventricular myocardium [4]. Due to the reduced size of the functioning right ventricle and some degree of tricuspid regurgitation, the contractility of the right ventricle is impaired, resulting in a decrease of effective output from the right side of the heart. The right ventricle function can worsen over time if a volume load causes dilatation and decreases the contractility even further [5]. Most cases of the Ebstein anomaly are sporadic and the genetic etiology is largely unknown, although mutations in several genes have been shown to be associated with this condition [6,7,8,9]. Overall, it is believed that genetic, environmental, and reproductive factors are instrumental in the development of Ebstein anomaly [1,2,3,10]. As the anatomical features of Ebstein anomaly can have varying degrees of severity, the clinical course can also vary widely. In mild cases, patients can be asymptomatic, while in severe cases, marked cyanosis, significant cardiomegaly, and signs of heart failure can be present right after birth [11,12]. Ebstein anomaly is frequently associated with other cardiac malformations and, in rarer instances, with non-cardiac malformations or genetic syndromes. The most common of cardiac malformations are interatrial communications—patent oval foramen and atrial septal defect, being present in up to 79–89% of patients [13,14]. Besides interatrial communications, other cardiac defects are found in 35 to 39% of patients with Ebstein anomaly [2,13,15,16]. Associated cardiac anomalies include pulmonary stenosis, pulmonary atresia, ventricular septal defect, cardiomyopathies, coarctation of aorta, bicuspid aortic valve, mitral valve prolapse, and other rarer abnormalities [2,13,15,16,17,18,19]. Among associated cardiac anomalies, right aortic arch is extremely rare and only few such cases have been described in the literature so far [1,20,21,22]. Non-cardiac malformations or genetic syndromes are found in about 19–22% of patients with Ebstein anomaly. Ebstein anomaly has been found among patients with Trisomy 21, Trisomy 9, Trisomy 13, Fragile X syndrome, Noonan syndrome, and other syndromes [1,2]. Several cases of Ebstein anomaly in patients with Charge syndrome have also been reported in the literature, but such association is rather rare and Ebstein anomaly represents less than 1% of all cardiac defects seen in patients with Charge syndrome [23]. In this case report, we present a rare combination of Ebstein anomaly and right aortic arch in a patient with Charge syndrome, which, to the best of the authors’ knowledge, is the first such case reported in the literature so far.

## 2. Case Report

We present a case of a five-year-old female who was diagnosed with Ebstein anomaly and right aortic arch prenatally at 20 weeks of gestation by fetal echocardiography. The pregnancy was conceived via in vitro fertilization by 37 years old nullipara woman. Up to 35 weeks of gestation, the mother of the patient used nadroparin and acetylsalicylic acid as a treatment for a thrombophilia with protein S deficiency. There was no other remarkable family history and all previous standard pregnancy follow ups were unremarkable.

**Figure 1:**
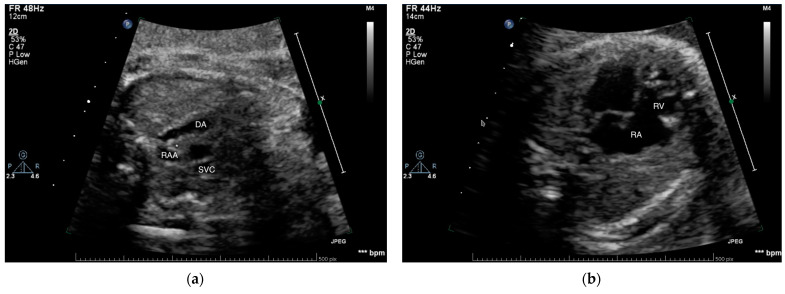
Images of fetal echocardiography at 20 weeks of gestation: (**a**) Transverse gray-scale sonogram of the fetal thorax showing aortic arch (RAA) coursing to the right of trachea (*), ductus arteriosus (DA) and superior vena cava (SVC), (**b**) transverse gray-scale sonogram of the fetal heart showing enlarged right atrium (RA), small right ventricle (RV), and apically placed dysplastic tricuspid valve.

As pregnancy was conceived via in vitro fertilization and thus regarded high risk, the woman was referred to our medical center (Children’s Clinical University Hospital in Riga, Latvia) for fetal echocardiography at 20 weeks of gestation, resulting in the diagnosis of Ebstein anomaly and right-sided aortic arch. The tricuspid valve was noted to be dysplastic and apically displaced with mild regurgitation. The size of the right ventricle was decreased, and aortic arch was coursing to the right of trachea (Figure 1). Further follow-up visits were done once a month up to the delivery, with no remarkable changes in the condition.

The patient was born at 40 weeks of gestation in spontaneous vaginal delivery weighing 3450 g, having a length of 55 cm and an Apgar score of 9/9. At day 4, patient was transferred to our medical center for further evaluation. Physical exam revealed acrocyanosis and systolic murmur, findings of other organ systems were without any significant deviations from the norm.

Chest x-ray, electrocardiogram, Holter monitoring, transthoracic echocardiography, abdominal ultrasonography, neurosonography, karyotyping, and genetic testing for 22q11.2 deletion syndrome were done. Transthoracic echocardiography confirmed the prenatal diagnosis of Ebstein anomaly and right-sided aortic arch. The electrocardiogram showed a partial right bundle branch block. Other examinations and tests done were unremarkable. At day 11, patient was discharged home in an overall compensated state without any recommendations for pharmacotherapy.

**Figure 2:**
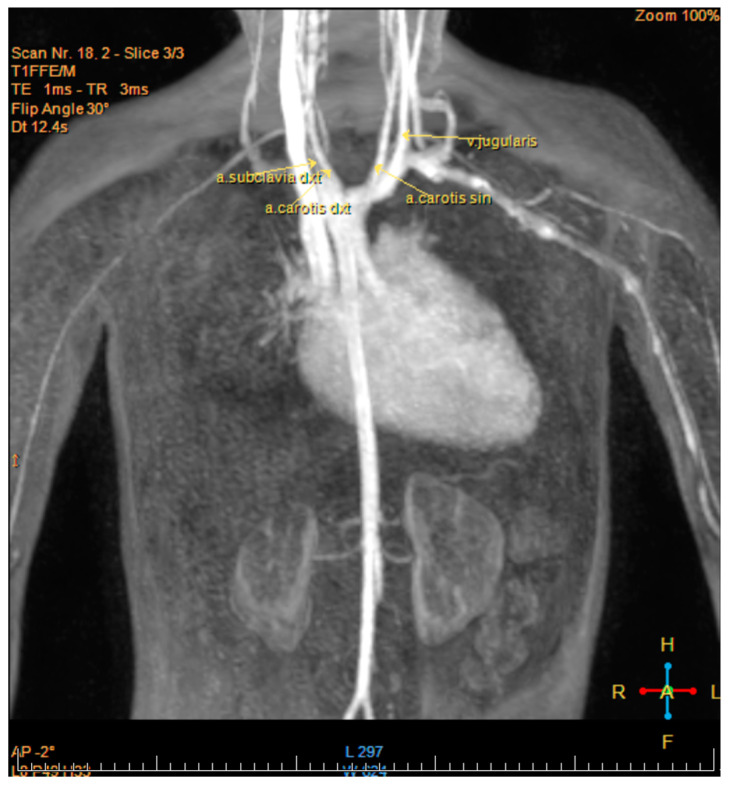
Cardiac magnetic resonance imaging (MRI) showing right aortic arch.

During the first six months of life, monthly follow up visits were done. The patient’s parents had no complaints and imaging studies did not show a significant progression of the condition. Further regular follow up visits with a pediatric cardiologist were done 1–2 times a year. The cardiac function in the first two years of life remained stable. Persistent oval foramen was observed, but it closed spontaneously at the age of three. Starting from the third year of life, progression of the condition was observed. At the age of four, due to progressive dilation of the right atrium and increasing regurgitation of the tricuspid valve, a magnetic resonance imaging was done to better assess the heart’s anatomy and aid in the consideration of the surgical treatment options. The magnetic resonance imaging showed that aortic arch and descending aorta in the mediastinum were located on the right side of the trachea in accordance with the previous diagnosis (see Figure 2). It also showed the tricuspid valve placed 23 mm apically from the mitral valve, moderate tricuspid regurgitation, and decreased ejection fracture of the right ventricle.

Although karyotyping and genetic testing for 22q11.2 deletion syndrome (DiGeorge syndrome) were done right after birth and were normal, overtime, some physical, motor, and mental development delays were observed and thus additional evaluation by medical genetic was done. It revealed some external ear anomalies, significant growth retardation (both weight and height below 3rd percentile), which together with congenital heart defects warranted more detailed genetic testing. The genetic test results revealed mutation in the chromodomain helicase DNA binding protein 7 (CHD7) gene, which, together with clinical features, allowed to establish a diagnosis of Charge syndrome.

## 3. Discussion

This case report deals with an extremely rare cardiac malformation combination, where Ebstein anomaly and right aortic arch are present in a patient with CHD7 positive Charge syndrome. To the best of the authors’ knowledge, thus far, no reports of a combination of both Ebstein anomaly and right aortic arch in a patient with Charge syndrome have been described in the literature.

Charge syndrome is a rare genetic disorder characterized by a combination of congenital anomalies (coloboma of the eye, heart defects, atresia of the choanae, retardation of growth, genital abnormalities, and ear abnormalities) mainly caused by de novo mutations in CHD7 gene [24,25,26,27]. In our patient, external ear anomalies, heart and aortic arch defects, and retardation of growth were present. The cardiac defect is one of the key characteristics of the syndrome and, as our case also highlights, may be one of the most significant determinants of the patients’ clinical course. The phenotype of cardiac malformations is highly variable and both isolated and combined heart defects are seen in patients with Charge syndrome. The most common cardiac malformations are conotruncal, septal, and atrioseptal defects. Aortic arch defects are also common and are found in 14–41% of patients with Charge syndrome that have heart involvement. One of the aortic arch defects found in patients with Charge syndrome is right aortic arch [26,27,28,29,30].

Right aortic arch is a rare congenital defect of the aorta, in which an aortic arch courses to the right of the trachea instead of the left [31,32,33]. Based on the pattern of the origin of the aortic arch branches, several types of right aortic arch can be distinguished [34,35]. The type most commonly associated with cardiac malformations is the mirror image branching type. Research shows that 77–100% of patients with mirror image branching type also have intracardiac malformations [34,36,37,38]. Moreover, in our reported case the patient had the right aortic arch of the mirror image branching type. In the mirror image branching type, the left innominate artery arises as the first branch of the aorta, followed by the right common carotid and the right subclavian arteries as the second and third branches, respectively [35]. The position of ductus arteriosus can also vary. The possible variants include the absence of it, it being positioned on the right connecting descending aorta with right pulmonary artery, or it being positioned on the left connecting either descending aorta or branch vessel to the left pulmonary artery [38]. In most cases, the right aortic arch of mirror image branching type does not create a vascular ring and thus does not cause any symptoms [33]. However, around 22% of patients with the right aortic arch of mirror image branching type have ductus arteriosus positioned on the left connecting descending aorta with the left pulmonary artery. Such arrangement creates a true vascular ring and thus can cause symptoms in some patients due to the compression of the trachea and/or the esophagus [39,40]. The symptoms may include stridor, recurrent upper respiratory tract infections, respiratory distress, cough, dysphagia, nausea, and vomiting [31,37,41]. In our reported case, the vascular ring was not formed, and thus patient did not exhibit any of the aforementioned symptoms.

Despite the fact that, in most cases, patients with mirror branching type right aortic arch do not experience any symptoms, the presence of right aortic arch still can have important clinical implications if surgical treatment is necessary for coexisting cardiac or other malformations [12]. It may be especially important if the surgical management plan includes placement of the modified Blalock Taussig shunt, in which a synthetic graft is placed from the innominate or the subclavian artery to the pulmonary artery [42]. The modified Blalock Taussig shunt is sometimes used as a part of surgical treatment in severely symptomatic neonates with Ebstein anomaly [43]. In cases where aortic arch courses on the right, the construction of the modified Blalock Taussig shunt may be difficult and thus application of further modifications or even alternative surgical methods may be needed [20,44].

This case highlights the significance of examining the position of the aortic arch and pattern of its branches in addition to other echocardiographic parameters in Ebstein’s anomaly as it may, in some instances, explain unaccounted symptoms as well as have important implications on the decision of surgical management strategy, especially in severe cases of Ebstein anomaly. In addition, if both Ebstein anomaly and right aortic arch are present, a patient should be thoroughly examined to look for other congenital anomalies and also genetic syndromes such as Charge should be considered, especially if over time retardation of growth is observed.

## Data Availability

Not applicable.
